# 1807. Implications of Sewer Connectivity in the U.S. for Equity in Wastewater-Based Epidemiology

**DOI:** 10.1093/ofid/ofad500.1636

**Published:** 2023-11-27

**Authors:** QinQin Yu, Scott Olesen, Claire Duvallet, Yonatan H Grad

**Affiliations:** Harvard T.H. Chan School of Public Health, Boston, Massachusetts; Biobot Analytics, Washington, District of Columbia; Biobot Analytics, Washington, District of Columbia; Harvard Chan School of Public Health, Boston, Massachusetts

## Abstract

**Background:**

Wastewater-based epidemiology is a promising public health tool that can yield a more representative view of the population than case reporting. However, only about 80% of the U.S. population is connected to public sewers, and the characteristics of populations missed by wastewater-based epidemiology are unclear.

**Methods:**

We used publicly available datasets to assess sewer connectivity in the U.S. by location, demographic groups, and economic groups. We used mathematical modeling to assess the impact of wastewater sampling inequities on inferences about epidemic trajectories at a local scale.

**Results:**

Data from the U.S. Census’ American Housing Survey (AHS) revealed that sewer connectivity was lower than average for American Indian and Alaskan Native, White, non-Hispanic, older, non-poverty, and larger households. Data from the U.S. Census’ American Community Survey and Environmental Protection Agency (EPA) revealed geographical areas with low sewer connectivity, such as Alaska, the Navajo Nation, Minnesota, Michigan, Florida. In contrast to the AHS data, data from the EPA showed that sewer connectivity was correlated with income in Minnesota, Florida, and California. However, with the exception of the U.S. Census data, there were inconsistencies across datasets.

Using mathematical modeling, we found that even weak connections between communities may allow wastewater monitoring in one community to serve as a reliable proxy for an interacting community of similar population size with no wastewater monitoring when cases are widespread.

**Conclusion:**

A systematic, rigorous assessment of variation in sewer connectivity will be important for ensuring an equitable and informed implementation of wastewater-based epidemiology as a public health monitoring system.

**Disclosures:**

**Scott Olesen, PhD**, Biobot Analytics: Former employee **Claire Duvallet, PhD**, Biobot Analytics: Former employee|Biobot Analytics: Stocks/Bonds **Yonatan H. Grad, MD, PhD**, Day Zero Diagnostics: Board Member|GSK: Advisor/Consultant

